# Genetic Polymorphisms of Multidrug Resistance Gene-1 (*MDR1/ABCB1*) and Glutathione S-Transferase Gene and the Risk of Inflammatory Bowel Disease among Moroccan Patients

**DOI:** 10.1155/2015/248060

**Published:** 2015-10-28

**Authors:** Nezha Senhaji, Yaya Kassogue, Mina Fahimi, Nadia Serbati, Wafaa Badre, Sellama Nadifi

**Affiliations:** ^1^Laboratory of Genetics and Molecular Pathologies, Faculty of Medicine and Pharmacy of Casablanca, Casablanca, Morocco; ^2^Gastroenterology Department, CHU Ibn Rochd, Casablanca, Morocco

## Abstract

Inflammatory bowel diseases (IBD) are multifactorial disorders resulting from environmental and genetic factors. Polymorphisms in* MDR1* and* GSTs* genes might explain individual differences in susceptibility to IBD. We carried out a case-control study to examine the association of* MDR1* (C1236T and C3435T),* GSTT1*, and* GSTM1* polymorphisms with the risk of IBD. Subjects were genotyped using PCR-RFLP for* MDR1* gene and multiplex PCR for* GSTT1* and* GSTM1*. Meta-analysis was performed to test the association of variant allele carriage with IBD risk. We report that* GSTT1* null genotype is significantly associated with the risk of CD (OR: 2.5, CI: 1.2–5, *P* = 0.013) and UC (OR: 3.5, CI: 1.5–8.5, *P* = 0.004) and can influence Crohn's disease behavior. The interaction between* GSTT1* and* GSTM1* genes showed that the combined null genotypes were associated with the risk of UC (OR: 3.1, CI: 1.1–9, *P* = 0.049). Furthermore, when compared to combined 1236CC/CT genotypes, the 1236TT genotype of* MDR1* gene was associated with the risk of UC (OR: 3.7, CI: 1.3–10.7, *P* = 0.03). Meta-analysis demonstrated significantly higher frequencies of 3435T carriage in IBD patients. Our results show that* GSTT1* null and* MDR1* polymorphisms could play a role in susceptibility to IBD.

## 1. Introduction

Inflammatory bowel disease (IBD) is a multifactorial disorder of the gastrointestinal tract including Crohn's disease (CD) and ulcerative colitis (UC). Although considerable progress has been made in the field of IBD research, the underlying etiopathogenesis is still under investigation [1]. It is assumed that inappropriate immune response to commensal intestinal bacteria associated with defective mucosal barrier related to genetic and environmental factors might play a fundamental role in the onset of IBD [2, 3]. The involvement of oxidant/antioxidant imbalance in the development and severity of IBD is well documented. Previous studies have demonstrated the role of candidate genes such as the multidrug resistance 1 (*MDR1*) and glutathione S-transferases (GSTs) in protecting cells against toxins, xenobiotics, or their metabolites [4]. The* MDR1* gene encodes a member of the ABC transporter subfamily B, a transmembrane P-glycoprotein (P-gp) of 170 kDa, which functions as an adenosine triphosphate-dependent efflux transporter pump [5]. P-gp is highly expressed on the apical surfaces of superficial columnar epithelial cells of the colon and distal small bowel. High levels are also found in small biliary ductules and small pancreatic ductules [6]. The high constitutive levels of P-gp expression in the gut suggest a role as a protective barrier against the absorption of endogenous or exogenous toxins and possibly a putative role in modulation of host-bacterial interactions [7, 8]. Among the polymorphisms identified in* MDR1* gene, the most widely investigated in IBD association studies as well as in other diseases are the 1236C>T (exon 12; rs1128503, Gly412Gly), 2677G>T/A (exon 21; rs2032582, Ala893Ser/Thr), and 3435C>T (exon 26; rs1045642, Ile1145Ile) with conflicting results in different populations around the world [9–11].

GSTs are phase II xenobiotic metabolizing enzymes. They play a critical role in cellular protection against reactive electrophiles and fatty acid hydroperoxides generated by oxidative stress through the conjugation with reduced glutathione. Therefore, GSTs facilitate the detoxification of cells by limiting tissue damage from free radical attack [12, 13].* GSTM1* (GST-mu 1) and* GSTT1* (GST-theta 1) located on chromosomes 1p13.3 and 22q11.2, respectively, are two members of the GSTs family being most frequently studied [14, 15]. Common deletion variants (termed null) of the structural* GSTM1* and* GSTT1* genes are associated with either decreased or impaired enzyme function [16]. Several studies have demonstrated the association of* GSTM1* and* GSTT1* genes with the risk of various cancers including bladder, gastric, and oral cancers and chronic myeloid leukemia [17–20]. However, few studies have addressed the relationship between* GSTM1* and* GSTT1* polymorphisms and the susceptibility of inflammatory and autoimmune diseases such as IBD [21–23]. To the best of our knowledge, the relationship between polymorphisms in* MDR1* and* GSTs* genes and the risk of IBD has not been examined so far in the Moroccan population. Therefore, our study investigated the role of* GSTM1*,* GSTT1*,* MDR1* C1236T, and C3435T SNPs in determining disease susceptibility in Moroccan patients.

## 2. Materials and Methods

### 2.1. Study Population

A total of 110 patients diagnosed with IBD at the Department of Gastroenterology, CHU Ibn Rochd (Casablanca, Morocco), were selected. Blood samples from 100 blood donors were used as controls. The diagnosis of CD or UC was established according to conventional clinical, endoscopic, radiological, and histological criteria as previously described [24, 25]. Patient's clinical and demographic characteristics were collected in a case report form (Supplementary Files) (see Supplementary Material available online at http://dx.doi.org/10.1155/2015/248060). The local ethics committee approved the study and a written informed consent was obtained from all participants.

### 2.2. Genotyping of* MDR1*,* GSTM1*, and* GSTT1* Polymorphisms

Genomic DNA was extracted from whole blood using the salting-out method. DNA concentration and quality were analyzed using a NanoDrop 1000 spectrophotometer (Thermo Fisher Scientific, Wilmington, DE, USA). Genotyping of C1236T and C3435T SNPs was done by polymerase chain reaction, restrictive fragment length polymorphism. The primer sequences, enzymatic restriction conditions, and digestion product sizes were previously described [26, 27]. To identify the genotypes of* GSTM1* and* GSTT1*, a multiplex polymerase chain reaction (PCR) was performed, in which* BCL2* gene was used as an internal control. PCR amplification condition and products sizes were previously described [28].

### 2.3. Meta-Analysis

#### 2.3.1. Inclusion and Exclusion Criteria

Genetic association studies were included in our meta-analysis if they met the following criteria: (a) a case-control study design, (b) studies that evaluated the association between the* MDR1* C3435T, C1236T,* GSTM1*, and* GSTT1* polymorphisms and IBD, and (c) the study reporting sufficient data to calculate allele frequencies and odds ratios of cases and controls for carriage of* MDR1* 3435T and 1236T alleles. Major exclusion criteria were (a) case-only study and review articles and (b) studies without raw data of the* MDR1* and GST genotypes.

#### 2.3.2. Pooled Studies for Case-Control Meta-Analysis

Twenty-seven case-control studies investigating* MDR1* polymorphisms in IBD patients were identified through the literature search. Eighteen studies that met the inclusion criteria were retrieved in* MDR1 C3435T* meta-analysis (Table 1). Five of them were included in* MDR1 C1236T* meta-analysis (Table 2).* GSTM1* and* GSTT1* meta-analysis reported data from 3 of the included studies with 789 cases and 792 controls (Table 3). The risk of IBD associated with the reported polymorphisms was estimated for each study by odds ratio (OR) and 95% confidence interval (95% CI). The meta-ORs were estimated using a fixed-effects model. Genetic heterogeneity was tested by Cochran's (*Q*) test and *I* ^2^ statistics was used to quantify the between-study heterogeneity effect. When a significant *Q* test (*Q* > 0.10; *I* ^2^ > 50%) indicated heterogeneity across studies, data were recombined using a random-effects model to estimate common ORs. The meta-analyses were conducted by MedCalc v.11.6.1.0 software.

### 2.4. Statistical Analysis

Data analysis has been carried out using the statistical package SPSS version 16 (SPSS Inc., Chicago, IL, USA). Differences in distribution of demographic and clinical features of patients with respect to their genotypes were done by chi-square test or Fisher Exact Test. The same tests were used to compare the distribution of genotypes between patients and controls as well as assess the Hardy-Weinberg Equilibrium in MDR1 gene. Odds ratio (OR) with a confidence interval (CI) of 95% was calculated to measure the strength of association between C1236T, MDR1 C3435T, GSTT1, and GSTM1 and the risk of inflammatory bowel disease. A *P* value less than 0.05 was considered as statistically significant.

## 3. Results

The present case-control study reports the frequencies of MDR1 C1236T, MDR1 C3435T, GSTT1, and GSTM1 in 77 CD patients, 33 UC patients, and 100 unrelated healthy controls. The two SNPs of MDR1 did not deviate from Hardy-Weinberg Equilibrium in control subjects. Allele and genotype frequencies of C1236T and C3435T polymorphisms in patients and controls are summarized in Table 4. The distribution of genotype frequencies showed no influence on the risk of CD and UC (*P* > 0.05). This finding remained valid for the allele frequencies (*P* > 0.05). In Table 5, the consideration of the recessive model showed that carriers of 1236TT genotypes were more exposed to developing UC when compared to the combined 1236CC/CT genotype (OR: 3.7, CI: 1.3–10.7, *P* = 0.03). However, the dominant model showed no particular effect whatever the type of considered SNP is. As presented in Table 6, we found that* GSTM1* null genotype frequency was higher in CD patients without being statistically significant (OR 1.2, CI: 0.6–2.1, *P* > 0.05). The same trend was observed in UC patients (OR: 1.5, CI: 0.7–3.3, *P* > 0.05). Surprisingly, we noticed that the* GSTT1* null genotype was significantly associated with the risk of CD (OR: 2.5, CI: 1.2–5, *P* = 0.013) and the risk of UC (OR: 3.5, CI: 1.5–8.5, *P* = 0.004). Furthermore, the interaction between* GSTM1* and* GSTT1* showed that the combined null genotype (*GSTM1* null,* GSTT1* null) was associated with the risk of UC at the limit of the statistical level (OR 3.1, CI: 1.1–9, *P* = 0.049) (Table 7). The distribution of demographic and clinical features of CD and UC patients with respect to genotypes of* GSTM1*, C1236T (exon 12), and C3435T (exon 26) in* MDR1* gene showed no particular trend of association (data not shown). On the other hand, frequency of the stricturing form was statistically higher in CD patients carrying the* GSTT1* null genotype compared to the penetrating or inflammatory forms (52% versus 33.3%; 13.6%, *P* = 0.02). However, the association between smoking status (current, former, or never smoking) and CD/UC was not influenced by genetic polymorphisms in genes encoding the GSTs metabolizing enzymes (Table 8).

**Figure 1:**
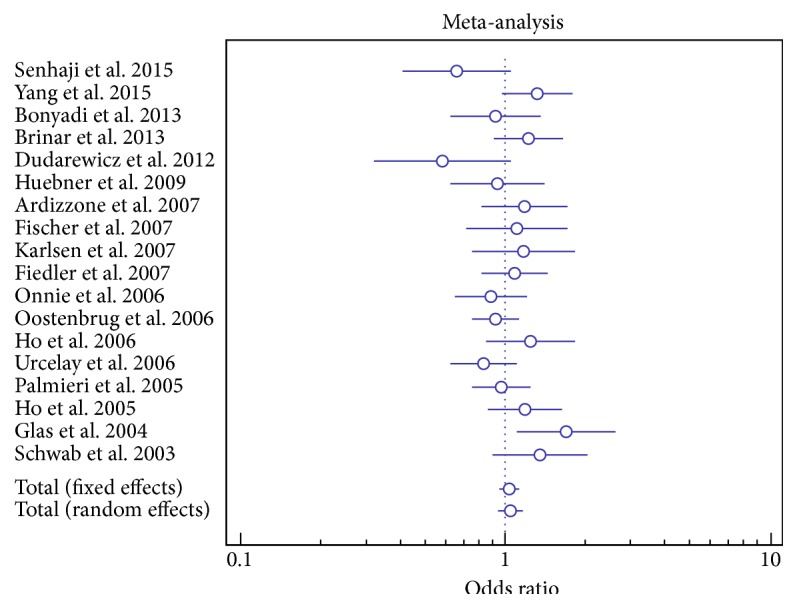
Forest plots for the association of MDR1 C3435T polymorphism and risk of IBD.

Based on the studies published on* MDR1* C3435T combined to our results, we observed a significant association between the T allele and IBD risk (Table 1, Figure 1). Meta-analysis of our dataset with the published studies on C1236T showed an overall protective effect of the variant allele (Table 2). On the other hand, when combining the very few results on* GSTM1* and* GSTT1* (Table 3), a significant heterogeneity in frequencies of the null genotype distribution in IBD patients was reported (*GSTM1* test for heterogeneity: *Q* = 28.95; DF = 2; *I* ^2^ = 93.1%; *P* < 0.0001;* GSTT1* test for heterogeneity: *Q* = 61.47; DF = 2; *I* ^2^ = 97%; *P* < 0.0001).

## 4. Discussion

In the present case-control study, we investigated the potential influence of* MDR1* C1236T,* MDR1* C3435T,* GSTM1*, and* GSTT1* polymorphisms on the risk of CD and UC disease. No genotype-phenotype correlation was observed between clinical characteristics of patients and the different genotypes of C1236T and C3435T polymorphisms in* MDR1* (data not shown). Consistent with our results, Fischer et al. reported a lack of association between C3435T and IBD phenotype in Hungarian patients [29]. In contrast, Huebner et al. found that CD behavior was influenced by the C3435T SNP [30]. We noted that the null genotype of* GSTT1* was higher in CD patients with the stricturing form. In contrast to our findings, Karban et al. reported that neither* GSTM1* null nor the* GSTT1* null genotypes were found to be associated with CD or UC phenotypes [21]. The frequencies of* MDR1* C1236T and* MDR1* C3435T in our patients and controls were statically comparable (*P* > 0.05). Similar findings for the C3435T SNP were reported by Wang et al., in a meta-analysis based essentially on Caucasians [31]. Brinar et al. found that the C3435T was associated with the risk of UC, while the heterozygous 3435CT was associated with a protective effect against CD in Croatian population [32]. Furthermore, Juyal et al. have demonstrated that the C1236T was significantly associated with susceptibility to UC, particularly in the earlier age of onset [33]. It is noteworthy that in our study the recessive model of* MDR1* C1236T was statistically associated with the risk of UC (*P* < 0.03). The discrepancy between our findings and those studies might be explained not only by the difference in minor allele distribution of* MDR1* C1236T and* MDR1* C3435T in our population but also by the relative small sample size of the present study. We have previously reported the existence of linkage disequilibrium between C1236T and C3435T in* MDR1* gene in our population [26]. It is well established that haplotypes consideration is more informative in association studies [34, 35]. However, in this study we were not able to perform haplotypes analysis due to the relative small sample size. Several studies have provided evidence that the C3435T SNP is associated with IBD and the results of our meta-analysis consolidated this variant as a potential risk factor for IBD in different populations. It is noteworthy that a lack of heterogeneity between studies was observed regarding distribution of both* MDR1* polymorphisms. On the other hand, we have interestingly noted that the* GSTT1* null genotype was found to be significantly associated with the risk of both CD (OR: 2.5, *P* = 0.013) and UC disease (OR: 3.5, *P* = 0.004). Consistent with our finding, Mittal et al. have reported a significant association of* GSTT1* null genotype with susceptibility to CD and UC in Indian population; however, they also found an association of* GSTM1* null genotype with the risk of UC that we could not replicate [23]. Karban et al. showed that* GSTT1* and* GSTM1* were associated with the risk of CD and UC in Israeli population [21]. The interaction between the two genes in our study showed that the combined* GSTM1* null/*GSTT1* null genotype was associated with the risk of UC at the limit of statistical level. Both previously reported studies have shown the association of the double deletion in* GSTM1* and* GSTT1* genes with IBD [21, 23]. A correlation between* GSTs* polymorphisms and IBD has been rarely discussed; more association studies are needed to validate the conclusions.

## 5. Conclusion

This is the first study to examine the association of* MDR1*,* GSTM1*, and* GSTT1* polymorphisms with the risk of IBD in a sample of the Moroccan population. It follows from the present case-control study that* GSTT1* null genotype and* MDR1* C1236T in the recessive model are associated with the risk of IBD. Crohn's disease behavior was influenced by the* GSTT1* null genotype. Moreover, the combined null genotype of* GSTT1* and* GSTM1* was associated with the risk of UC with a limited effect.

## Supplementary Material

Genotypic distribution of MDR1 polymorphisms according to Crohn's disease and Ulcerative colitis patient's demographic and clinical characteristics are presented in Supplementary Table 1 and 2 respectively. In addition, genotypic distributions of GSTM1 and GSTT1 with respect to demographic and clinical characteristics of Crohn's disease and Ulcerative colitis patients are presented in Supplementary Table 3 and 4 respectively.

## Figures and Tables

**Table 1:** Pooled analysis of studies exploring the role of *MDR1* C3435T in IBD.

Study	Cases (event/total)	Controls (events/total)	Odds ratio	95% CI
Senhaji et al. 2015 (the current paper)	75/220	53/120	0,65	0,41 to 1,03
Yang et al. 2015 [36]	121/298	152/446	1,32	0,97 to 1,79
Bonyadi et al. 2013 [37]	120/232	99/184	0,92	0,62 to 1,35
Brinar et al. 2013 [32]	304/612	106/238	1,22	0,91 to 1,66
Dudarewicz et al. 2012 [38]	77/108	111/137	0,58	0,32 to 1,05
Huebner et al. 2009 [30]	600/759	156/195	0,94	0,63 to 1,39
Ardizzone et al. 2007 [39]	180/288	123/210	1,17	0,81 to 1,69
Fischer el al. 2007 [29]	308/414	106/146	1,09	0,71 to 1,67
Karlsen et al. 2007 [40]	231/268	310/368	1,16	0,74 to 1,82
Fiedler et al. 2007 [41]	304/388	782/1015	1,07	0,81 to 1,43
Onnie et al. 2006 [9]	1071/1408	219/280	0,88	0,65 to 1,20
Oostenbrug et al. 2006 [42]	757/1420	293/530	0,92	0,75 to 1,12
Ho et al. 2006 [43]	352/428	205/260	1,24	0,84 to 1,83
Urcelay et al. 2006 [44]	405/614	227/324	0,82	0,61 to 1,10
Palmieri et al. 2005 [45]	697/946	335/450	0,96	0,74 to 1,24
Ho et al. 2005 [46]	486/603	288/370	1,18	0,86 to 1,62
Glas et al. 2004 [47]	213/258	195/265	1,69	1,11 to 2,59
Schwab et al. 2003 [10]	216/275	201/275	1,34	0,91 to 1,99
Total (fixed effects)	6517/9539	3961/5813	1,03	0,96 to 1,12
Total (random effects)	6517/9539	3961/5813	1,04	0,94 to 1,15

Test for heterogeneity: *Q* = 26.12; DF = 17; *I* ^2^ = 35%; *P* = 0.07.

**Table 2:** Pooled analysis of studies exploring the role of *MDR1* C1236T in IBD.

Study	Cases (event/total)	Controls (events/total)	Odds ratio	95% CI
Senhaji et al. 2015 (the current paper)	75/220	65/200	1,07	0,71 to 1,61
Yang et al. 2015 [36]	181/300	275/446	0,94	0,70 to 1,27
Huebner et al. 2009 [30]	686/1554	181/398	0,94	0,75 to 1,18
Oostenbrug et al. 2006 [42]	610/1420	228/530	0,99	0,81 to 1,22
Ho et al. 2006 [43]	400/856	236/522	1,06	0,85 to 1,32
Total (fixed effects)	1952/4350	985/2096	0,99	0,89 to 1,11
Total (random effects)	1952/4350	985/2096	0,99	0,89 to 1,11

Test for heterogeneity: *Q* = 0.78; DF = 4; *I* ^2^ = 0%; *P* = 0.94.

**Table 3:** Pooled analysis of studies exploring the role of *GSTM1* and *GSTT1* in IBD.

Study	Cases (event/total)	Controls (events/total)	Odds ratio	95% CI
Studies on *GSTM1*				
Senhaji et al. 2015 (the current paper)	62/110	51/100	1,24	0,72 to 2,13
Karban et al. 2011 [21]	277/574	300/528	0,70	0,55 to 0,89
Mittal et al. 2007 [23]	61/105	49/164	3,25	1,95 to 5,42
Total (fixed effects)	400/789	400/792	0,97	0,80 to 1,18
Total (random effects)	400/789	400/792	1,39	0,55 to 3,53

Studies on *GSTT1*				
Senhaji et al. 2015 (the current paper)	40/110	17/100	2,79	1,45 to 5,34
Karban et al. 2011 [21]	172/574	97/528	1,90	1,43 to 2,52
Mittal et al. 2007 [23]	95/105	26/164	50,42	23,23 to 109,4
Total (fixed effects)	307/789	140/792	3,13	2,49 to 3,95
Total (random effects)	307/789	140/792	6,26	1,06 to 36,87

**Table 4:** Distribution of genotypes and alleles of MDR1 polymorphisms in IBD patients and controls.

Genotypes/alleles	Control	CD	OR	*P* value	UC	OR	*P* value
	*N* (%)	*N* (%)	(95% CI)		*N* (%)	(95% CI)	
1236CC	43 (43)	33 (43)	Ref.		12 (36)	Ref.	
1236CT	49 (49)	42 (55)	1.1 (0.6–2)	0.75	13 (39)	1 (0.4–2.3)	1
1236TT	8 (8)	2 (3)	0.3 (0.01–0.6)	0.2	8 (24)	3.6 (1–11.5)	**0.05**
1236C	135 (67.5)	108 (70)	Ref.		37 (56)	Ref.	
1236T	65 (32.5)	46 (30)	0.9 (0.6–0.4)	0.65	29 (44)	1.6 (1–2.9)	0.1
HWE p	0.24	0.01			0.29		

3435CC	39 (39)	30 (39)	Ref.		16 (48)	Ref.	
3435CT	51 (51)	40 (52)	1 (0.5–0.9)	1	13 (39)	0.6 (0.3–1.4)	0.3
3435TT	10 (10)	7 (9)	0.9 (0.3–2.7)	1	4 (12)	1 (0.3–3.6)	1
3435C	129 (64.5)	100 (65)	Ref.		45 (68)	Ref.	
3435T	71 (35.5)	54 (35)	1 (0.6–1.5)	1	21 (32)	0.8 (0.5–1.5)	0.65
HWE p	0.25	0.32			0.69		

HWE: Hardy-Weinberg Equilibrium; CD: Crohn disease; UC: ulcerative colitis; OR: odds ratio; *N*: number; CC: homozygous wild type; CT: heterozygous; TT: homozygous variant.

**Table 5:** Distribution of genetic models of MDR1 polymorphisms in IBD patients and controls.

Genotypes/alleles	Control	CD	OR	*P* value	UC	OR	*P* value
	*N* (%)	*N* (%)	(95% CI)		*N* (%)	(95% CI)	
1236CC/CT^a^	92 (92)	75 (97.5)	Ref.		25 (76)	Ref.	
1236TT	8 (8)	2 (2.5)	0.3 (0.06–1.5)	0.2	8 (24)	3.7 (1.3–10.7)	**0.03**
1236CC^b^	43 (43)	33 (43)	Ref.		12 (36)	Ref.	
1236CT/TT	57 (57)	44 (57)	1 (0.5–1.8)	1	21(64)	1.3 (0.6–2.9)	0.5

3435CC/CT^a^	90 (90)	70 (91)	Ref.		29 (88)	Ref.	
3435TT	10 (10)	7 (9)	1 (0.3–2.5)	1	4 (12)	1.2 (0.4–4.3)	0.7
3435CC^b^	39 (39)	30 (39)	Ref.		16 (48)	Ref.	
3435CT/TT	61 (61)	47 (61)	1 (0.5–1.8)	1	17 (52)	0.7 (0.31–1.5)	0.42

^a^Recessive model; ^b^dominant model; CD: Crohn disease; UC: ulcerative colitis; OR: odds ratio; *N*: number; CC: homozygous wild type; CT: heterozygous; TT: homozygous variant.

**Table 6:** Frequencies of GSTM1 and GSTT1 polymorphisms between IBD patients and controls.

Genotypes	Control	CD	OR	*P *	UC	OR	*P *
	*N* (%)	*N* (%)	95% CI		*N* (%)	95% CI	
*GSTM1*							
Present	49 (49)	35 (45.5)	Ref.		13 (39.4)	Ref.	
Null	51 (51)	42 (54.5)	1.2 (0.6–2.1)	0.65	20 (60.6)	1.5 (0.7–3.3)	0.42
*GSTT1*							
Present	83 (83)	51 (66.2)	Ref.		19 (57.6)	Ref.	
Null	17 (17)	26 (33.8)	2.5 (1.2–5)	**0.013**	14 (42.4)	3.5 (1.5–8.5)	**0.004**

**Table 7:** Risk assessment of IBD, regarding different combinations of *GSTM1* and *GSTT1* genotypes.

*GSTM1*	*GSTT1*	Control	CD	OR	*P *	UC	OR	*P*
		*N* (%)	*N* (%)	95% CI		*N* (%)	95% CI	
Present	Present	45 (45)	27 (35.1)	Ref.		9 (27.3)	Ref.	
Present	Null	4 (4)	8 (10.4)	3.3 (1–12.12)	0.1	4 (12.1)	5 (1–23.8)	0.05
Null	Present	35 (35)	24 (31.2)	1.1 (0.6–2.3)	0.7	10 (30.3)	1.4 (0.5–3.9)	0.6
Null	Null	16 (16)	18 (23.4)	1.8 (0.8–4.3)	**0.15**	10 (30.3)	3.1 (1.1–9)	**0.049**

**Table 8:** Genotypic distribution of *GSTM1* and *GSTT1* with respect to smoking status and CD behavior.

Parameters	*GSTM1* null	*GSTM1* present	*χ* ^2^	*P*	*GSTT1* null	*GSTT1* present	*χ* ^2^	*P*
CD behavior *n* (%)								
Inflammatory	14 (63.6)	8 (36.4)	4.2	0.12	3 (13.6)	19 (86.4)	7.7	**0.02**
Stricturing	16 (64)	9 (36)			13 (52)	12 (48)		
Penetrating	12 (40)	18 (60)			10 (33.3)	20 (66.7)		
Smoking status *N* (%)								
Crohn's disease patients								
Yes (current/former)	15 (55.6)	12 (44.4)	0.02	1	9 (33.3)	18 (66.7)	0.003	1
No (never)	27 (54)	23 (46)			17 (34)	33 (66)		
Ulcerative colitis patients								
Yes (current/former)	7 (77.8)	2 (22.2)	1.5	0.26	4 (44.4)	5 (55.6)	0.02	1
No (never)	13 (54.2)	11 (45.8)			10 (41.7)	14 (58.3)		

*GSTM1* null: deleted glutathione S-transferase mu 1 gene; *GSTM1 *present: functional glutathione S-transferase mu 1 gene; *GSTT1 *null: deleted glutathione S-transferase theta 1 gene; *GSTT1 *present: functional glutathione S-transferase theta 1 gene.

## References

[1] M’Koma A. E. (2013). Inflammatory bowel disease: an expanding global health problem. *Clinical Medicine Insights: Gastroenterology*.

[2] Low D., Mizoguchi A., Mizoguchi E. (2013). DNA methylation in inflammatory bowel disease and beyond. *World Journal of Gastroenterology*.

[3] Frolkis A., Dieleman L. A., Barkema H. W. (2013). Environment and the inflammatory bowel diseases. *Canadian Journal of Gastroenterology*.

[4] Ekhart C., Rodenhuis S., Smits P. H. M., Beijnen J. H., Huitema A. D. R. (2009). An overview of the relations between polymorphisms in drug metabolising enzymes and drug transporters and survival after cancer drug treatment. *Cancer Treatment Reviews*.

[5] Ueda K., Pastan I., Gottesman M. M. (1987). Isolation and sequence of the promoter region of the human multidrug-resistance (P-glycoprotein) gene. *The Journal of Biological Chemistry*.

[6] Thiebaut F., Tsuruo T., Hamada H., Gottesman M. M., Pastan I., Willingham M. C. (1987). Cellular localization of the multidrug-resistance gene product P-glycoprotein in normal human tissues. *Proceedings of the National Academy of Sciences of the United States of America*.

[7] Marzolini C., Paus E., Buclin T., Kim R. B. (2004). Polymorphisms in human *MDR1* (P-glycoprotein): recent advances and clinical relevance. *Clinical Pharmacology and Therapeutics*.

[8] Fromm M. F. (2002). Genetically determined differences in P-glycoprotein function: implications for disease risk. *Toxicology*.

[9] Onnie C. M., Fisher S. A., Pattni R. (2006). Associations of allelic variants of the multidrug resistance gene (ABCB1 or MDR1) and inflammatory bowel disease and their effects on disease behavior: a case-control and meta-analysis study. *Inflammatory Bowel Diseases*.

[10] Schwab M., Schaeffeler E., Marx C. (2003). Association between the C3435T *MDR1* gene polymorphism and susceptibility for ulcerative colitis. *Gastroenterology*.

[11] Gazouli M., Zacharatos P., Gorgoulis V., Mantzaris G., Papalambros E., Ikonomopoulos I. (2004). The C3435T MDR1 gene polymorphism is not associated with susceptibility for ulcerative colitis in Greek population. *Gastroenterology*.

[12] Rezaie A., Parker R. D., Abdollahi M. (2007). Oxidative stress and pathogenesis of inflammatory bowel disease: an epiphenomenon or the cause?. *Digestive Diseases and Sciences*.

[13] Hayes J. D., McLellan L. I. (1999). Glutathione and glutathione-dependent enzymes represent a co-ordinately regulated defence against oxidative stress. *Free Radical Research*.

[14] Pearson W. R., Vorachek W. R., Xu S.-J. (1993). Identification of class-mu glutathione transferase genes GSTM1-GSTM5 on human chromosome 1p13. *The American Journal of Human Genetics*.

[15] Webb G., Vaska V., Coggan M., Board P. (1996). Chromosomal localization of the gene for the human Theta class glutathione transferase (GSTT1). *Genomics*.

[16] Hallier E., Langhof T., Dannappel D. (1993). Polymorphism of glutathione conjugation of methyl bromide, ethylene oxide and dichloromethane in human blood: influence on the induction of sister chromatid exchanges (SCE) in lymphocytes. *Archives of Toxicology*.

[17] Sharma T., Jain S., Verma A. (2013). Gene environment interaction in urinary bladder cancer with special reference to organochlorine pesticide: a case control study. *Cancer Biomarkers*.

[18] Ma W., Zhuang L., Han B., Tang B. (2013). Association between glutathione S-transferase T1 null genotype and gastric cancer risk: a meta-analysis of 48 studies. *PLoS ONE*.

[19] Dong G., Tian Y., Chen S., Xu X., Zheng J., Li T. (2013). Glutathione S-transferase T1 null genotype is associated with oral cancer susceptibility in Asian populations. *Tumor Biology*.

[20] Bhat G., Bhat A., Wani A. (2012). Polymorphic variation in glutathione-S-transferase genes and risk of chronic myeloid leukaemia in the Kashmiri population. *Asian Pacific Journal of Cancer Prevention*.

[21] Karban A., Krivoy N., Elkin H. (2011). Non-jewish Israeli IBD patients have significantly higher glutathione S-transferase GSTT1-null frequency. *Digestive Diseases and Sciences*.

[22] Duncan H., Swanx C., Green J. (1995). Susceptibility to ulcerative colitis and Crohn’s disease: interactions between glutathione S-transferase GSTM1 and GSTT1 genotypes. *Clinica Chimica Acta*.

[23] Mittal R. D., Manchanda P. K., Bid H. K., Ghoshal U. C. (2007). Analysis of polymorphisms of tumor necrosis factor-*α* and polymorphic xenobiotic metabolizing enzymes in inflammatory bowel disease: study from northern India. *Journal of Gastroenterology and Hepatology*.

[24] Malchow H., Ewe K., Brandes J. W. (1984). European Cooperative Crohn’s Disease Study (ECCDS): results of drug treatment. *Gastroenterology*.

[25] Senhaji N., Diakité B., Serbati N., Zaid Y., Badre W., Nadifi S. (2014). Toll-like receptor 4 Asp299Gly and Thr399Ile polymorphisms: new data and a meta-analysis. *BMC Gastroenterology*.

[26] Kassogue Y., Dehbi H., Nassereddine S., Quachouh M., Nadifi S. (2013). Genotype variability and haplotype frequency of MDR1 (ABCB1) gene polymorphism in Morocco. *DNA and Cell Biology*.

[27] Yaya K., Hind D., Meryem Q., Asma Q., Said B., Sellama N. (2014). Single nucleotide polymorphisms of multidrug resistance gene 1 (*MDR1*) and risk of chronic myeloid leukemia. *Tumor Biology*.

[28] Kassogue Y., Quachouh M., Dehbi H., Quessar A., Benchekroun S., Nadifi S. (2014). Effect of interaction of glutathione *S*-transferases (T1 and M1) on the hematologic and cytogenetic responses in chronic myeloid leukemia patients treated with imatinib. *Medical Oncology*.

[29] Fischer S., Lakatos P. L., Lakatos L. (2007). ATP-binding cassette transporter ABCG2 (BCRP) and ABCB1 (MDR1) variants are not associated with disease susceptibility, disease phenotype response to medical therapy or need for surgeryin Hungarian patients with inflammatory bowel diseases. *Scandinavian Journal of Gastroenterology*.

[30] Huebner C., Browning B. L., Petermann I. (2009). Genetic analysis of MDR1 and inflammatory bowel disease reveals protective effect of heterozygous variants for ulcerative colitis. *Inflammatory Bowel Diseases*.

[31] Wang J., Guo X., Yu S. (2014). MDR1 C3435T polymorphism and inflammatory bowel disease risk: a meta-analysis. *Molecular Biology Reports*.

[32] Brinar M., Cukovic-Cavka S., Bozina N. (2013). *MDR1* polymorphisms are associated with inflammatory bowel disease in a cohort of Croatian IBD patients. *BMC Gastroenterology*.

[33] Juyal G., Midha V., Amre D., Sood A., Seidman E., Thelma B. K. (2009). Associations between common variants in the *MDR1* (ABCB1) gene and ulcerative colitis among North Indians. *Pharmacogenetics and Genomics*.

[34] Hoffmeyer S., Burk O., von Richter O. (2000). Functional polymorphisms of the human multidrug-resistance gene: multiple sequence variations and correlation of one allele with P-glycoprotein expression and activity in vivo. *Proceedings of the National Academy of Sciences of the United States of America*.

[35] Vivona D., Bueno C. T., Lima L. T. (2012). ABCB1 haplotype is associated with major molecular response in chronic myeloid leukemia patients treated with standard-dose of imatinib. *Blood Cells, Molecules, & Diseases*.

[36] Yang Q., Chen B., Zhang Q. (2015). Contribution of *MDR1* gene polymorphisms on IBD predisposition and response to glucocorticoids in IBD in a Chinese population. *Journal of Digestive Diseases*.

[37] Bonyadi M. J., Gerami S. M., Somi M. H., Khoshbaten M. (2013). Effect of the C3435T polymorphism of the multidrug resistance 1 gene on the severity of inflammatory bowel disease in Iranian Azeri Turks. *Saudi Journal of Gastroenterology*.

[38] Dudarewicz M., Barańska M., Rychlik-Sych M., Trzciński R., Dziki A., Skreţkowicz J. (2012). C3435T polymorphism of the ABCB1/MDR1 gene encoding P-glycoprotein in patients with inflammatory bowel disease in a Polish population. *Pharmacological Reports*.

[39] Ardizzone S., Maconi G., Bianchi V. (2007). Multidrug resistance 1 gene polymorphism and susceptibility to inflammatory bowel disease. *Inflammatory Bowel Diseases*.

[40] Karlsen T. H., Hampe J., Wiencke K. (2007). Genetic polymorphisms associated with inflammatory bowel disease do not confer risk for primary sclerosing cholangitis. *The American Journal of Gastroenterology*.

[41] Fiedler T., Büning C., Reuter W. (2007). Possible role of MDR1 two-locus genotypes for young-age onset ulcerative colitis but not Crohn’s disease. *European Journal of Clinical Pharmacology*.

[42] Oostenbrug L. E., Dijkstra G., Nolte I. M. (2006). Absence of association between the multidrug resistance (*MDR1*) gene and inflammatory bowel disease. *Scandinavian Journal of Gastroenterology*.

[43] Ho G.-T., Soranzo N., Nimmo E. R., Tenesa A., Goldstein D. B., Satsangi J. (2006). ABCB1/MDR1 gene determines susceptibility and phenotype in ulcerative colitis: discrimination of critical variants using a gene-wide haplotype tagging approach. *Human Molecular Genetics*.

[44] Urcelay E., Mendoza J. L., Martín M. C. (2006). MDR1 gene: susceptibility in Spanish Crohn’s disease and ulcerative colitis patients. *Inflammatory Bowel Diseases*.

[45] Palmieri O., Latiano A., Valvano R. (2005). Multidrug resistance 1 gene polymorphisms are not associated with inflammatory bowel disease and response to therapy in Italian patients. *Alimentary Pharmacology & Therapeutics*.

[46] Ho G.-T., Nimmo E. R., Tenesa A. (2005). Allelic variations of the multidrug resistance gene determine susceptibility and disease behavior in ulcerative colitis. *Gastroenterology*.

[47] Glas J., Török H.-P., Schiemann U., Folwaczny C. (2004). *MDR1* gene polymorphism in ulcerative colitis. *Gastroenterology*.

