# Supplementary material for: Genetic Polymorphisms of Multidrug Resistance Gene-1 (MDR1/ABCB1) and Glutathione S-Transferase Gene and the Risk of Inflammatory Bowel Disease among Moroccan Patients
Source: Mediators Inflamm. 2015 Oct 28;2015:248060. doi: 10.1155/2015/248060 (PMC4641206; doi:10.1155/2015/248060)
Supplement: Supplementary file 1 — Genotypic distribution of MDR1 polymorphisms according to Crohn's disease and Ulcerative colitis patient's demographic and clinical characteristics are presented in Supplementary Table 1 and 2 respectively. In addition, genotypic distributions of GSTM1 and GSTT1 with respect to demographic and clinical characteristics of Crohn's disease and Ulcerative colitis patients are presented in Supplementary Table 3 and 4 respectively. [file 248060.f1.docx]

**Table 1:** Genotypic distribution of C1236T and C3435T polymorphisms according to Crohn disease patient’s demographic and clinical characteristics

| Parameters | C1236T N (%) | | | X^2^ | P | C3435T N (%) | | | X^2^ | P |
| --- | --- | --- | --- | --- | --- | --- | --- | --- | --- | --- |
|  | CC | CT | TT |  |  | CC | CT | TT |  |  |
| Gender N (%) |  |  |  |  |  |  |  |  |  |  |
| Female | 12 (50) | 11 (45.8) | 1 (4.2) | 1.23 | 0.5 | 12 (50) | 10 (41.7) | 2 (8.3) | 1.82 | 0.4 |
| Male | 21 (39.6) | 31 (58.5) | 1 (1.9) |  |  | 18 (34) | 30 (56.6) | 5 (9.4) |  |  |
| Age (years) |  |  |  |  |  |  |  |  |  |  |
| ≤16 | 2 (50) | 2 (50) | - | 1.63 | 0.8 | 3 (75) | 1 (25) | - | 6.7 | 0.15 |
| 17-40 | 20 (39.2) | 29 (56.9) | 2 (3.9) |  |  | 15 (29.4) | 30 (58.8) | 6 (11.8) |  |  |
| > 40 | 11 (50) | 11 (50) | - |  |  | 12 (54.6) | 9 (40.9) | 1 (4.5) |  |  |
| Localization N (%) | |  |  |  |  |  |  |  |  |  |
| Ileum (L1±L4) | 10 (41.7) | 14 (58.3) | - | 6.27 | 0.4 | 9 (37.5) | 12 (50) | 3 (12.5) | 6.9 | 0.32 |
| Colon (L2±L4) | 10 (45.5) | 10 (45.5) | 2 (9) |  |  | 9 (40.9) | 9 (40.9) | 4 (18.2) |  |  |
| Ileo-colon (L3±L4) | 13 (43.3) | 17 (56.7) | - |  |  | 12 (40) | 18 (60) | - |  |  |
| UGI (L4) | - | 1 (100) | - |  |  | - | 1 (100) | - |  |  |
| Behavior n (%) |  |  |  |  |  |  |  |  |  |  |
| Inflammatory | 8 (36.4) | 13 (59.1) | 1 (4.5) | 1.7 | 0.78 | 11 (50) | 8 (36.4) | 3 (13.6) | 6.1 | 0.2 |
| Stricturing | 11 (44) | 13 (52) | 1 (4) |  |  | 9 (36) | 16 (64) | - |  |  |
| Penetrating | 14 (46.7) | 16 (53.3) | - |  |  | 10 (33.3) | 16 (53.4) | 4 (13.3) |  |  |
| EIM N (%) | | |  |  |  |  |  |  |  |  |
| Yes | 16 (42.1) | 22 (57.9) | - | 2.1 | 0.34 | 19 (50) | 16 (42.1) | 3 (7.9) | 3.86 | 0.14 |
| No | 17 (43.6) | 20 (51.3) | 2 (5.1) |  |  | 11 (28.2) | 24 (61.5) | 4 (10.3) |  |  |
| Family history N (%) | |  |  |  |  |  |  |  |  |  |
| Yes | 3 (75) | 1 (25) | - | 1.8 | 0.4 | 3 (75) | 1 (25) | - | 2.4 | 0.3 |
| No | 30 (41.1) | 41 (56.2) | 2 (2.7) |  |  | 27 (37) | 39 (53.4) | 7 (9.6) |  |  |
| Smoking history N (%) | |  |  |  |  |  |  |  |  |  |
| Yes | 8 (29.6) | 18 (66.7) | 1 (3.7) | 3.01 | 0.22 | 9 (33.3) | 16 (59.3) | 2 (7.4) | 0.8 | 0.64 |
| No | 25 (50) | 24 (48) | 1 (2) |  |  | 21 (42) | 24 (48) | 5 (10) |  |  |
| Surgery N (%) |  |  |  |  |  |  |  |  |  |  |
| Yes | 17 (44.7) | 21 (55.3) | - | 2.02 | 0.36 | 15 (39.5) | 20 (52.6) | 3 (7.9) | 0.13 | 0.9 |
| No | 16 (41) | 21 (53.9) | 2 (5.1) |  |  | 15 (38.5) | 20 (51.3) | 4 (10.2) |  |  |

EIM: extra intestinal manifestations; N: number; CC: wild type; CT: heterozygous; TT: homozygous variant

**Table 2:** Genotypic distribution of C1236T and C3435T polymorphisms according to ulcerative colitis patient’s demographic and clinical characteristics

| Parameters | C1236T N (%) | | | X^2^ | P | C3435T N (%) | | | X^2^ | P |
| --- | --- | --- | --- | --- | --- | --- | --- | --- | --- | --- |
|  | CC | CT | TT |  |  | CC | CT | TT |  |  |
| Gender N (%) |  |  |  |  |  |  |  |  |  |  |
| Female | 6 (40) | 5 (33.3) | 4 (26.7) | 0.42 | 0.8 | 8 (53.4) | 5 (33.3) | 2 (13.3) | 0.8 | 0.8 |
| Male | 6 (33.3) | 8 (44.5) | 4 (22.2) |  |  | 8 (44.4) | 8 (44.4) | 2 (11.1) |  |  |
| Age (years) |  |  |  |  |  |  |  |  |  |  |
| 17-40 | 5 (31.2) | 6 (37.5) | 5 (31.2) | 0.88 | 0.64 | 7 (43.8) | 8 (50) | 1 (6.2) | 1.9 | 0.4 |
| > 40 | 7 (41.2) | 7 (41.2) | 3 (17.6) |  |  | 9 (52.9) | 5 (29.4) | 3 (17.6) |  |  |
| Disease extention N (%) | |  |  |  |  |  |  |  |  |  |
| E1 | 3 (75) | - | 1 (25) | 6.9 | 0.33 | 3 (75) | 1 (25) | - | 4.2 | 0.65 |
| E2 | 6 (46.2) | 4 (30.7) | 3 (23.1) |  |  | 7 (53.8) | 5 (38.5) | 1 (7.7) |  |  |
| E3 | - | 2 (66.7) | 1 (33.3) |  |  | 1 (33.3) | 2 (66.7) | - |  |  |
| E4 | 3 (23.1) | 7 (53.8) | 3 (23.1) |  |  | 5 (38.5) | 5 (38.5) | 3 (23) |  |  |
| EIM N (%) | | |  |  |  |  |  |  |  |  |
| Yes | 8 (44.4) | 7 (38.9) | 3 (16.7) | 1.65 | 0.44 | 8 (44.4) | 8 (44.4) | 2 (11.1) | 0.42 | 0.81 |
| No | 4 (26.7) | 6 (40) | 5 (33.3) |  |  | 8 (53.3) | 5 (33.3) | 2 (13.3) |  |  |
| Family history N (%) | |  |  |  |  |  |  |  |  |  |
| Yes | - | - | 1 (100) | 3.22 | 0.2 | - | 1 (100) | - | 1.6 | 0.45 |
| No | 12 (37.5) | 13 (40.6) | 7 (21 .9) |  |  | 16 (50) | 12 (37.5) | 4 (12.5) |  |  |
| Smoking history N (%) | |  |  |  |  |  |  |  |  |  |
| Yes | 3 (33.3) | 4 (44.4) | 2 (22.2) | 0.13 | 0.9 | 4 (44.4) | 4 (44.4) | 1 (11.1) | 0.13 | 0.9 |
| No | 9 (37.5) | 9 (37.5) | 6 (25) |  |  | 12 (50) | 9 (37.5) | 3 (12.5) |  |  |
| Surgery N (%) |  |  |  |  |  |  |  |  |  |  |
| Yes | 2 (33.3) | 2 (33.3) | 2 (33.3) | 0.33 | 0.84 | 2 (33.3) | 4 (66.7) | - | 2.6 | 0.27 |
| No | 10 (37) | 11 (40.7) | 6 (22.2) |  |  | 14 (51.9) | 9 (33.3) | 4 (14.8) |  |  |

EIM: extra intestinal manifestations; N: number; CC: wild type; CT: heterozygous; TT: homozygous variant

**Table 3:** Genotypic distribution of GSTM1 and GSTT1 with respect to demographic and clinical characteristics in Crohn disease patients

| Parameters | *GSTM1 null* | *GSTM1 present* | X^2^ | P | *GSTT1*  *Null* | *GSTT1 present* | X^2^ | P |
| --- | --- | --- | --- | --- | --- | --- | --- | --- |
| Gender N (%) |  |  |  |  |  |  |  |  |
| Female | 13 (54.2) | 11 (45.8) | 0.002 | 0.5 | 9 (37.5) | 15 (62.5) | 0.22 | 0.8 |
| Male | 29 (54.7) | 24 (45.3) |  |  | 26 (33.8) | 51 (66.2) |  |  |
| Age (years) |  |  |  |  |  |  |  |  |
| ≤16 | 3 (75) | 1 (25) | 1.12 | 0.57 | 1 (25) | 3 (75) | 0.22 | 0.9 |
| 17-40 | 26 (51) | 25 (49) |  |  | 18 (35.3) | 33 (64.7) |  |  |
| > 40 | 13 (59.1) | 9 (40.9) |  |  | 7 (31.8) | 15 (68.2) |  |  |
| Localisation N (%) | |  |  |  |  |  |  |  |
| Ileum (L1±L4) | 13 (54.2) | 11 (45.8) | 3.42 | 0.33 | 7 (29.2) | 17 (70.8) | 0.92 | 0.8 |
| Colon (L2±L4) | 9 (40.9) | 13 (59.1) |  |  | 8 (36.4) | 14 (63.6) |  |  |
| Ileo-colon (L3±L4) | 19 (63.3) | 11 (36.7) |  |  | 11 (36.7) | 19 (63.3) |  |  |
| UGI (L4) | 1 (100) | - |  |  | - | 1 (100) |  |  |
| Behavior n (%) |  |  |  |  |  |  |  |  |
| Inflammatory | 14 (63.6) | 8 (36.4) | 4.2 | 0.12 | 3 (13.6) | 19 (86.4) | 7.7 | **0.02** |
| Stricturing | 16 (64) | 9 (36) |  |  | 13 (52) | 12 (48) |  |  |
| Penetrating | 12 (40) | 18 (60) |  |  | 10 (33.3) | 20 (66.7) |  |  |
| EIM N (%) | | |  |  |  |  |  |  |
| Yes | 17 (44.7) | 21 (55.3) | 2.9 | 0.11 | 10 (26.3) | 28 (73.7) | 1.86 | 0.23 |
| No | 25 (64.1) | 14 (35.9) |  |  | 16 (41) | 23 (59) |  |  |
| Family history N (%) | |  |  |  |  |  |  |  |
| Yes | 2 (50) | 2 (50) | 0.03 | 1 | 1 (25) | 3 (75) | 0.15 | 1 |
| No | 40 (54.8) | 33 (45.2) |  |  | 25 (34.2) | 48 (65.8) |  |  |
| Smoking history N (%) | |  |  |  |  |  |  |  |
| Yes | 15 (55.6) | 12 (44.4) | 0.02 | 1 | 9 (33.3) | 18 (66.7) | 0.003 | 1 |
| No | 27 (54) | 23 (46) |  |  | 17 (34) | 33 (66) |  |  |
| Surgery N (%) |  |  |  |  |  |  |  |  |
| Yes | 20 (51.3) | 19 (48.7) | 0.33 | 0.65 | 15 (38.5) | 24 (61.5) | 0.7 | 0.4 |
| No | 22 (57.9) | 16 (42.1) |  |  | 11 (28.9) | 27 (71.1) |  |  |

EIM: extra intestinal manifestations; N: number; *GSTM1 null*: deleted glutathione S-transferase mu 1 gene; *GSTM1* present: functional glutathione S-transferase mu 1 gene; *GSTT1 null*: deleted glutathione S-transferase teta 1 gene; *GSTT1* present: functional glutathione S-transferase teta 1 gene.

**Table 4:** Genotypic distribution of GSTM1 and GSTT1 regarding the demographic and clinical characteristics of ulcerative colitis patients

| Parameters | GSTM1 null | GSTM1 present | X^2^ | P | GSTT1 null | GSTT1 present | X^2^ | P |
| --- | --- | --- | --- | --- | --- | --- | --- | --- |
| Gender N (%) |  |  |  |  |  |  |  |  |
| Female | 6 (40) | 9 (60) | 4.9 | 0.04 | 5 (33.3) | 10 (66.7) | 0.9 | 0.5 |
| Male | 14 (77.8) | 4 (22.2) |  |  | 9 (50) | 9 (50) |  |  |
| Age (years) |  |  |  |  |  |  |  |  |
| 17-40 | 12 (75) | 4 (25) | 2.7 | 0.16 | 6 (37.5) | 10 (62.5) | 0.3 | 0.7 |
| > 40 | 8 (47.1) | 9 (52.9) |  |  | 8 (47.1) | 9 (52.9) |  |  |
| Disease extention N (%) | |  |  |  |  |  |  |  |
| E1 | 3 (75) | 1 (25) | 3.4 | 0.33 | 1 (25) | 3 (75) | 0.7 | 0.86 |
| E2 | 8 (61.5) | 5 (38.5) |  |  | 6 (46.2) | 7 (53.8) |  |  |
| E3 | 3 (100) | - |  |  | 1 (33.3) | 2 (66.7) |  |  |
| E4 | 6 (46.2) | 7 (53.8) |  |  | 6 (46.2) | 7 (53.8) |  |  |
| EIM N (%) | | |  |  |  |  |  |  |
| Yes | 12 (66.7) | 6 (33.3) | 0.6 | 0.4 | 8 (44.4) | 10 (55.6) | 0.06 | 1 |
| No | 8 (53.3) | 7 (46.7) |  |  | 6 (40) | 9 (60) |  |  |
| Family history N (%) | |  |  |  |  |  |  |  |
| Yes | - | 1 (100) | 1.6 | 0.4 | - | 1 (100) | 0.76 | 1 |
| No | 20 (62.5) | 12 (37.5) |  |  | 14 (43.8) | 18 (56.2) |  |  |
| Smoking history N (%) | |  |  |  |  |  |  |  |
| Yes | 7 (77.8) | 2 (22.2) | 1.5 | 0.26 | 4 (44.4) | 5 (55.6) | 0.02 | 1 |
| No | 13 (54.2) | 11 (45.8) |  |  | 10 (41.7) | 14 (58.3) |  |  |
| Surgery N (%) |  |  |  |  |  |  |  |  |
| Yes | 5 (83.3) | 1 (16.7) | 1.6 | 0.4 | 4 (44.4) | 5 (55.6) | 0.02 | 1 |
| No | 15 (55.6) | 12 (44.4) |  |  | 10 (41.7) | 14 (58.3) |  |  |

EIM: extra intestinal manifestations; N: number; *GSTM1 null*: deleted glutathione S-transferase mu 1gene; *GSTM1* present: functional glutathione S-transferase mu 1 gene; *GSTT1* null: deleted glutathione S-transferase teta 1 gene; *GSTT1* present: functional glutathione S-transferase teta 1 gene.
